# Simultaneous Removal of Mycotoxins by a New Feed Additive Containing a Tri-Octahedral Smectite Mixed with Lignocellulose

**DOI:** 10.3390/toxins14060393

**Published:** 2022-06-08

**Authors:** Donato Greco, Vito D’Ascanio, Mariagrazia Abbasciano, Elisa Santovito, Antonella Garbetta, Antonio F. Logrieco, Giuseppina Avantaggiato

**Affiliations:** Institute of Sciences of Food Production (ISPA), National Research Council (CNR), 70126 Bari, Italy; donato.greco@ispa.cnr.it (D.G.); vito.dascanio@ispa.cnr.it (V.D.); mariagrazia.abbasciano@ispa.cnr.it (M.A.); elisa.santovito@ispa.cnr.it (E.S.); antonella.garbetta@ispa.cnr.it (A.G.); antonio.logrieco@ispa.cnr.it (A.F.L.)

**Keywords:** mycotoxins, detoxifying agents, tri-octahedral smectites, lignocellulose-based materials, antioxidant activity, feed additives, multi-mycotoxin adsorption

## Abstract

Simultaneous removal of mycotoxins has been poorly addressed, and a limited number of studies have reported the efficacy of feed additives in sequestering a large spectrum of mycotoxins. In this study, a new mycotoxin-adsorbing agent was obtained by properly mixing a tri-octahedral smectite with a lignocellulose-based material. At a dosage of 1 mg mL^−1^, these materials simultaneously adsorbed frequently occurring mycotoxins and did not exert a cytotoxic effect on intestinal cells. Chyme samples obtained by a simulated GI digestion did not affect the viability of Caco-2TC7 cells as measured by the MTT test. In addition, the chyme of the lignocellulose showed a high content of polyphenols (210 mg mL^−1^ catechin equivalent) and good antioxidant activity. The properties of the individual constituents were maintained in the final composite, and were unaffected by their combination. When tested with a pool of seven mycotoxins at 1 µg mL^−1^ each and pH 5, the composite (5 mg mL^−1^) simultaneously sequestered AFB_1_ (95%), FB_1_ (99%), ZEA (93%), OTA (80%), T-2 (63%), and DON (22%). HT-2 adsorption did not occur. Mycotoxin adsorption increased exponentially as dosage increased, and occurred at physiological pH values. AFB_1_, ZEA and T-2 adsorption was not affected by pH in the range 3–9, whereas OTA and FB_1_ were adsorbed at pH values of 3–5. The adsorbed amount of AFB_1_, ZEA and T-2 was not released when pH rose from 3 to 7. FB_1_ and OTA desorption was less than 38%. Langmuir adsorption isotherms revealed high capacity and affinity for adsorption of the target mycotoxins. Results of this study are promising and show the potential of the new composite to remove mycotoxins in practical scenarios where several mycotoxins can co-occur.

## 1. Introduction

Most animal feeds marketed in the European Union (EU) can be contaminated with structurally different mycotoxins, although only a small percentage contain higher levels than permitted or recommended. The negative synergistic effects produced when different mycotoxins are simultaneously present in feed may be responsible for symptoms of mycotoxicosis which lead to detrimental effects on animal health as well as significant economic losses [1]. Dietary supplementation with non-nutritive mycotoxin-detoxifying agents (MDAs) represents the most practical and widely studied method to reduce the effects of mycotoxin exposure. In 2009, the use of MDAs as technological feed additives became officially allowed in the EU [2]. Among those, mycotoxin adsorbing agents are the most used and economic feed additives that act in the gastrointestinal tract, preventing, or reducing mycotoxicosis in livestock and, in some cases, the carryover of mycotoxins into animal products. EFSA released a review on MDAs that covered aspects such as mode of action, efficacy, and safety [3]. Most studies reviewed by EFSA deal with adsorption of mycotoxins by feed additives, and those currently reported in the literature still focus on the efficacy of adsorption of a specific mycotoxin. However, these studies are not applicable to a real-world situation where the coexistence of several mycotoxins in feed ingredients is a cause of concern. Simultaneous adsorption of mycotoxins has been poorly addressed, and few studies to have reported the efficacy of materials in sequestering mycotoxins from a multi-component system, such as buffered solutions or contaminated feeds. Therefore, a study on simultaneous removal of mycotoxins is required to obtain an adsorbent suitable for a real-world scenario.

Potential adsorbent materials for mycotoxin reduction in feeds include activated carbons, silicate minerals (bentonites and zeolites), complex indigestible carbohydrates (cellulose, polysaccharides in the cell walls of yeast and bacteria such as glucomannans, peptidoglycans), and others [3]. 

The largest and most complex class of mycotoxin binders are the silicate minerals such as bentonite, an adsorbent swelling clay belonging to the smectite group. The term ‘bentonite’ can be used as a synonym for both di-octahedral and tri-octahedral smectites whose structural differences are widely described in a scientific guidance issued by EFSA [4]. The use of a di-octahedral smectite (i.e., montmorillonite) as an additive for feed decontamination of aflatoxins was authorized by the EU in 2013 with the claim 1m558 [5]. However, montmorillonite clays are not equally efficient in adsorbing in vitro aflatoxins [6], meaning they are not indiscriminately able to counteract toxic effects of aflatoxins in vivo or to prevent the carry-over of metabolites in animal-derived products. In addition, numerous in vitro studies concerning the highly selective efficacy of bentonites in adsorbing aflatoxin B_1_ (AFB_1_) have indicated limited tendency of these minerals to adsorb structurally different mycotoxins [7,8,9,10,11]. Selective aflatoxin adsorption by bentonites has been overcome by chemical modifications resulting in enhanced hydrophobicity when structural load balancing cations are exchanged with heavy molecular amines [10,11]. Two recent studies proposed the use of tri-octahedral smectites as potential technological feed additives for reduction of mycotoxins [12,13]. No data are available regarding the ability of these minerals to simultaneously adsorb mycotoxins from multi-toxins in aqueous solutions or feeds. 

In addition to silicate minerals, different polymeric sources of dietary fibres have been used to protect animals from different toxic compounds including mycotoxins [14,15,16]. Micronized fibres obtained from different plant materials, i.e., cereals or legumes (wheat, barley, alfalfa, oats, pea hulls) are mainly composed of cellulose, hemicellulose, and lignin, and they have been tested as mycotoxin binders [1]. In vivo studies showed the protective effect exerted by wheat fibres in reducing the intestinal absorption of ochratoxin A (OTA) and its bioavailability in piglets and rats fed contaminated diets [14,15]. The efficacy of modified lignocellulose materials in adsorbing mycotoxins including OTA, T-2 toxin (T-2), deoxynivalenol (DON) and nivalenol (NIV) was recorded in a US Patent [17]. In a recent study, Greco et al. [18] selected as promising multi-mycotoxin biosorbents some agricultural by-products rich in undegradable fibres (lignin, cellulose) and flavonoids, such as grape, artichoke, and almond by-products and waste. In another study, Avantaggiato et al. [19] showed that a red-grape pomace (pulp and skin) simultaneously adsorbed in vitro mycotoxins belonging to different classes, i.e., AFB_1_, zearalenone (ZEA), OTA, and fumonisin B_1_ (FB_1_). The effectiveness of this by-product in reducing the intestinal absorption of target mycotoxins was confirmed in pigs through the urinary biomarker approach [20]. Recently, Adunphatcharaphon et al. [21] produced an acid-modified Durian peel which can be considered a promising material for multi-mycotoxin biosorption. As suggested by Greco et al. [18], it is likely that the high efficacy of some agricultural by-products in sequestering different mycotoxins requires an appropriate balancing of the organic components rather than the presence of a single substance. Further research is required to clarify the components of agricultural by-products that are involved in the biosorption of mycotoxins and to confirm their efficacy in vivo. 

In addition to mycotoxin adsorption efficacy, dietary fibres can improve animal welfare at various levels, being a potentially valuable source of phenolics with antioxidant activity. In this context, the use of lignin in animal feed has recently aroused the attention of many researchers due to its antioxidant and antimicrobial properties. Lignin, the second most abundant component within the plant after cellulose, is a complex insoluble polymer and it has highly heterogeneous composition varying within a given cell. Marketed as lignosulphonate, it is widely used in feed manufacturing as a pellet binder to increase the durability of the structure of pelleted feed [22]. Recent studies have suggested its application to promote animal growth and welfare in the absence of antibiotics, which cannot be used as growth promoters or for prophylaxis following their ban by the European Union [23]. Other studies have highlighted the antioxidant and prebiotic activity of purified lignin in monogastric animals, favoring growth of beneficial bacteria, controlling intestinal pathogens, and improving morphological structures in the intestines [24,25,26]. In addition, improvements in the performance of animals such as Holstein calves, broiler chickens and geese were observed [23,27]. A recent study suggests that lignin exerts a bacteriostatic effect on intestinal pathogens by inhibiting microtubule formation during cellular mitosis [28].

In this context, the aim of this study was to explore the feasibility of using certain materials poorly researched in their capacity as anti-mycotoxin agents, i.e., tri-octahedral smectites and lignocellulose-based materials, to prepare a composite for multi-mycotoxin removal. As far as we know, this is the first time that these materials have been assessed for their ability to adsorb simultaneously a large spectrum of mycotoxins. In particular, this study examined in vitro the efficacy of twelve smectites (including di- and tri-octahedral smectites) and six lignocellulose-based materials for adsorbing mycotoxins at physiological pH values of the GI tract. A smectite and a lignocellulose showing multi-mycotoxin adsorption efficacy were selected to prepare a composite. Cytotoxic effect on intestinal cells, antioxidant activity, and polyphenols content of the selected materials were determined after a simulated GI digestion. Selected materials were properly combined taking into account their biological and mycotoxin-adsorbing properties. The composite was then studied to assess the effect of medium pH, adsorbent dosage, and toxin concentration on the extent of adsorption using equilibrium isotherms. Target mycotoxins were chosen taking into account their prevalence in feeds and their known effects on livestock health, and for which regulatory guidelines about maximum tolerable levels in feed are available, i.e., AFB_1_, ZEA, OTA, FB_1_, DON, T-2 and HT-2. Mycotoxin concentrations were properly set to study the behavior of materials in adsorbing mycotoxins even in a cocktail, and to calculate adsorption parameters. These concentrations approached or were above the EU maximum levels or recommendations for mycotoxins in animal feeds.

## 2. Results and Discussion

### 2.1. Screening of Adsorbent Materials as Multi-Mycotoxin Detoxifying Agents 

Preliminary work was performed to study the effect of a cocktail of mycotoxins on adsorption and to select promising multi-mycotoxin-adsorbing materials. Experimental conditions of our previous adsorption studies [18,19] were used as a standard. Hence, twelve Na/Ca smectites (comprising seven di-octahedral smectites and five tri-octahedral smectites) and six lignocellulose-based materials were tested using a fixed amount of material (1 mg mL^−1^) and of mycotoxins (1 µg mL^−1^). Mycotoxins tested as a pool were AFB_1_, ZEA, OTA, FB_1_, and DON, and were dissolved in buffers at pH 3 and 7 to simulate gastric and intestinal pH values (Table 1). T-2 and HT-2 were not assayed in the preliminary study. 

As expected, excluding the bentonite labelled 2 (a Na di-octahedral smectite), most smectites showed more than 90% adsorption of AFB_1_ at both pH values, regardless of their structure (di- or tri-octahedral). The in vitro efficacy of bentonite clays, especially di-octahedral smectites (i.e., montmorillonite), as AFB_1_ binders has been widely reported in the literature. So far, only montmorillonite has been authorized as a technological feed additive for aflatoxin decontamination of feeds for ruminants, poultry and pigs [5]. However, all smectites do not adsorbing aflatoxins equally and can differ depending on their intrinsic properties [6]. Recently, it has been proven that the efficacy of di-octahedral smectites in sequestering aflatoxins depends on their physico-chemical and mineralogical properties, including geological origin [6]. Tri-octahedral smectites have been poorly evaluated as mycotoxin binders, including for aflatoxins [12,13]. This may be due to the relative abundance of di-octahedral smectites compared to tri-octahedral varieties. In accordance with the findings of our screening study, a report by Vila-Donat et al. [12] proved the ability of tri-octahedral smectites in sequestering AFB_1_ to be even better than that of bentonites with di-octahedral structures. 

Although in our experimental conditions, di- or tri-octahedral smectites did not seem to differ in sequestering AFB_1_, they differed substantially in binding mycotoxins other than AFB_1_, which could be due to their origin and physiochemical properties, i.e., cation exchange capacity, pore volume and expandability [1,29]. Four out of twelve smectites efficiently adsorbed ZEA (>70%), regardless of pH. Interestingly, all these bentonites belonged to the group of tri-octahedral smectites, which is in line with the study by Vila-Donat et al. [13] that found good ZEA adsorption values for these minerals. In addition to tri-octahedral smectites, only heat-activated bentonites and organophilic bentonites can bind ZEA, to some extent [30,31,32,33]. 

All bentonites (except the bentonite labelled 2) showed high efficacy (>80%) in removing FB_1_ from medium at pH 3. When recorded at pH 7, FB_1_ adsorption values dropped to less than 20%. Three tri-octahedral smectites bound about 26% of the toxin amount in solution. The effect of medium pH on FB_1_ adsorption has been observed in several adsorbents, including minerals and organic materials [3,18,19,21,34]. 

OTA was adsorbed by most bentonites but to a different extent depending on pH of the medium, being significantly high at gastric pH and negligible at intestinal pH. Eight out of twelve bentonites adsorbed more than 70% of OTA at pH 3. The highest adsorption values (≥80%) were recorded for three bentonites, all belonging to the tri-octahedral smectite group. At neutral medium pH, OTA adsorption was less than 25%. The effect of pH on OTA adsorption has been widely reported in the literature for different types of adsorbents, including clays [18,19,35,36].

In accordance with previous reports [37,38,39], DON was poorly adsorbed by all bentonites, including the tri-octahedral smectites. Vila-Donat et al. [13] also found negligible DON adsorption by tri-octahedral smectites. 

In conclusion, the screening of smectites for simultaneous removal of mycotoxins allowed the selection of sodium smectites with tri-octahedral structures (materials labelled 9–12 in Table 1) as promising multi-toxin adsorbents. With respect to di-octahedral smectites, the smectites with tri-octahedral structures contain lower Al_2_O_3_ and higher MgO contents, which leads to different layer charges and physicochemical properties [4]. It is well known that the properties of smectites change not only with the magnitude of the layer charge but also with its distribution throughout the layer, with the exchangeable cations and with their hydration status [6]. Thus, for octahedral charged smectites (such as the di-octahedral smectite montmorillonite), the negative charge is delocalized over surface oxygens so that only weak hydrogen bonds can form with interlayer water. For tetrahedral charged smectites (such as the tri-octahedral smectites, beidellite and saponite), the charge is more localized and stronger hydrogen bonds can form between surface oxygens and interlayer water [4]. These different distributions of interlayer charge, together with different hydration statuses, are responsible for the different physicochemical properties of smectites and for their adsorbing features, including mycotoxins uptake.

In addition to tri-octahedral smectites, lignocellulose-based materials were found quite effective for simultaneously adsorbing the target mycotoxins (Table 1), although they appeared less effective than clays. In particular, AFB_1_ adsorption was in the range 47–81% at pH 3, and 60–87% at pH 7. Regardless of pH, a significant ZEA adsorption was also recorded, comparable to that obtained by smectites. ZEA adsorption values were in the range 37–77% at pH 3 and 61–71% at pH 7. FB_1_ and OTA adsorptions were again influenced by pH, being high at pH 3 (19–39% for FB_1_ and 35–73% for OTA), and negligible at pH 7 (less than 8%). The pH of the medium influences the extent of FB_1_ and OTA adsorption, since it affects the degree of ionization of their molecules, and the surface charge of the lignocellulose-based materials, which are characterized by the presence of phenolic and carboxylic groups. According to previous studies using agricultural by-products as mycotoxin binders [18,19], lignocelluloses did not adsorb DON.

As indicated by the supplier of the lignocelluloses, the materials were high-purity lignin products prepared by a patented technology used industrially in conjunction with the manufacture of high-quality papers. The materials were provided in the form of fine light brown powders (with <200 µm particle size) and differed depending on the location of production facilities (India, USA and Canada), and plant feedstock (annual fibers such as wheat straw, sugarcane bagasse, and wood). They were chemically characterized by the supplier as mixtures of natural polyphenols, composed of medium-to small polymer aggregates, particularly rich in OH phenolics, but also containing aliphatic and carboxylic groups. The chemical composition of the lignocelluloses did not differ substantially, thus they acted similarly in sequestering mycotoxins. 

The ability of lignocelluloses in simultaneously adsorbing AFB_1_, ZEA, OTA and FB_1_ is in accordance with the findings of our previous reports showing that certain fibre-rich agricultural by-products, tested at medium-low dosages (1–10 mg mL^−1^), were able to sequester different mycotoxins [18,19]. All these studies confirm that food plants that are available in large quantities, or certain waste products from agricultural and industrial operations (such as the lignocelluloses assayed herein), particularly rich in undegradable fibres (lignin, cellulose), are potentially low-cost products for mycotoxin adsorption.

Under experimental conditions of the screening study, two materials (one from each group of organic and inorganic binders) were selected as promising MMDAs, since they simultaneously adsorbed more than 70% of three different mycotoxins. These materials were the tri-octahedral smectite labelled 9 (which exhibited mycotoxin adsorption values comparable to the other tri-octahedral smectites) and the lignocellulose-based material labelled 6. The latter was one of the best adsorbents in its group. Because multi-toxin adsorption by the selected smectite was higher than the lignocellulose, especially for FB_1_ uptake, the materials were combined in mixtures containing smectite as the main component. Several combinations of these materials were attempted to prepare mixtures with satisfying multi-mycotoxin adsorption features. These composites were assayed for mycotoxin adsorption as single components (data not shown). The product obtained by mixing the smectite and the lignocellulose in the weight ratio 70:30 was found to be the best combination. As expected, the mixing of the smectite with the lignocellulose yielded a composite with mycotoxin adsorption values slightly lower than the mineral clay. However, the lignocellulose possessed unique biological properties (antioxidant activity), which justifies its use (see Section 2.2). Choosing such a component with mycotoxin-adsorbtion properties also had the effect of limiting the decrease in effectiveness for the best performing adsorbent (the smectite) due to its dilution in the mixture. 

The composite with the smectite/cellulose weight ratio of 70:30 was tested at 1 mg mL^−1^ of dosage with a multi-mycotoxin working solution containing 1 µg mL^−1^ of AFB_1_, FB_1_, ZEA, OTA, DON, T-2, and HT-2; and at pH 3, 5 and 7. With respect to the preliminary study for the screening of MMDAs, T-2 and HT-2 were included in the adsorption assay. The latter was performed at pH 5, being the physiological pH value of the duodenal compartment of monogastric animals, where main absorption of mycotoxins takes place. As shown in Table 2, the mixture retained the efficacy of its components in adsorbing simultaneously AFB_1_, FB_1_, ZEA and OTA, and showed a moderate efficacy (23–45%) in binding T-2. DON and HT-2 were poorly sequestered. AFB_1_, ZEA and T-2 adsorptions were unaffected by pH in the range 3–7, while FB_1_ and OTA adsorptions were higher at acid pH values. Interestingly, at pH 5 the new composite was still able to adsorb a high amount of FB_1_ (91%) and OTA (32%).

### 2.2. Evaluation of Cytotoxicity and Antioxidant Properties of Selected Multi-Mycotoxin Detoxifying Agents 

The aim of this study was to assess whether certain materials acting as mycotoxin adsorbents have biological effects (antioxidant properties) and can be used to fortify mycotoxin-detoxifying agents in preventing or counteracting toxic effects. Because the intestine system represents the specific and primary target of mycotoxin-contaminated feeds, the assessment of protective effect of mycotoxin-adsorbent materials was carried out on a cell line of this target organ (Caco-2TC7). The materials selected as MMDAs, i.e., the tri-octahedral smectite and the lignocellulose (items 9 and 6 in Table 1, respectively), were subjected to a gastro-intestinal digestion process and, subsequently, their chyme samples were analyzed for biological activity. Ascorbic acid was tested as a standard natural compound, as it is widely used in feed formulation as a preservative.

Prior to determining the antioxidant activity, a cytotoxic test was performed to assess the safety of the agents and to select the highest no-cytotoxic dilution of chyme samples. Cytotoxic assays were performed on the intestinal cell line Caco-2TC7 using the MTT test. A 1:10 dilution of the chyme samples, corresponding to 1 mg mL^−1^ of equivalent concentration of digested materials (tri-octahedral smectite and lignocellulose-based material) did not affect the viability of intestinal cells after 24 h of exposure. These results suggest that these materials did not exert toxic effects in vitro. On the contrary, in the same experimental conditions, the chyme sample of ascorbic acid at 1 mg mL^−1^ induced an 80% decrease in resorufin fluorescence, indicating cytotoxicity. 

The antioxidant activity of chyme samples was measured using the Cellular Antioxidant Activity (CAA) assay and was expressed as median effective dose (EC_50_), which is the concentration of the material (mg mL^−1^) that produces a 50% reduction of induced ROS. The lignocellulose-based material showed good antioxidant activity, although lower than ascorbic acid, with EC_50_ values at 0.248 ± 0.090 mg mL^−1^ and 0.008 ± 0.001 mg mL^−1^ of the digested material, respectively. The smectite did not show anti-oxidant properties up to 1 mg mL^−1^ of the digested product.

As reported by the supplier, the selected lignocellulose-based material consisted of phenylpropanoic polymer aggregates with a high grade of purity (about 95–97%) and high polyphenol content, as determined by the Folin-Ciocalteu assay and expressed as Gallic acid equivalent (mean value at 35 GAE/100 g of lignocellulose). These values are in accordance with data reported in the literature for analogous materials [40,41,42]. Because the lignocellulose contained a high level of polyphenols, it was supposed that they can be released during the digestion process and can determine biological properties of the ingested/digested material, including the antioxidant capacity. The lignocellulose was first digested, and the polyphenol content of the centrifuged chyme samples (relevant to 1 mg mL^−1^ of digested material) was measured by Folin-Ciocalteau assay and expressed as catechin equivalent. Interestingly, these chyme samples showed a high total content of polyphenols (210.9 ± 9.7 mg mL^−1^ of catechin equivalent), suggesting high bioaccessibility. The biological properties of polyphenols depend on their bioaccesibility, the process of releasing polyphenols from the food matrix in the GI through enzymatic hydrolysis, which may be at least partially absorbed. High bioaccessibility for total phenol content is of great importance since it is linked to the ability of these active compounds to counteract oxidative stress, pathogens, and infections.

### 2.3. Effect of pH on Mycotoxin Adsorption and Desorption Study 

The removal of mycotoxins from aqueous mediums through an adsorption process is, in most cases, dependent on pH value. The pH of the medium can affect the surface charge of adsorbents as well as the degree of ionization of toxins, and subsequently it can lead to a shift in reaction kinetics and equilibrium characteristics of the adsorption process. As reported by Greco et al. [18], it is important to underline that a good adsorbent should have high adsorption efficacy in a pH range similar to the one encountered along the gastrointestinal tract of monogastric animals (pH 1.5–7.5). Therefore, it should be able to bind mycotoxins at low pH (simulating the range of pH typical of the gastric compartment) and retain the bound toxins during the transit of the food bolus through the intestinal compartments.

**Figure 1:**
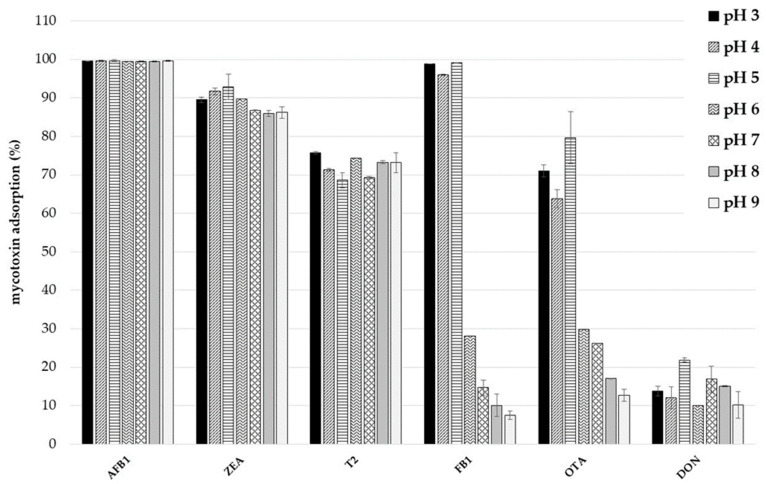
Effect of pH on multi-mycotoxin adsorption by a mixture containing a tri-octahedral smectite and a lignocellulose-based material. Adsorption trials were performed at different pH values, using an adsorbent dosage of 5 mg mL^−1^ and toxins concentration of 1 µg mL^−1^.

In this study, the effect of pH on toxin adsorption by the smectite–lignocellulose composite was investigated using a 5 mg mL^−1^ of dosage and a series of multi-mycotoxin solutions (1 µg mL^−1^) with pH values varying in the range 3–9. As shown in Figure 1, mycotoxin adsorptions differed according to the degree of ionization of toxins. AFB_1_, ZEA and T-2, which are mainly non-ionizable molecules, were adsorbed to the same extent regardless of pH, whereas FB_1_ and OTA were adsorbed mainly at acid pH. DON adsorption was negligible (<20%), while HT-2 adsorption was not recorded.

AFB_1_ is a planar, hydrophobic, and non-ionizable molecule, therefore a change of medium pH is not expected to affect its adsorption [43]. Indeed, AFB_1_ was completely sequestered (100%) from the mediums at all the assayed pH values. High ZEA adsorption values were also recorded (86–93%). ZEA is a weak acid with a pKa of 7.62 [19], it should be in the protonated (non-ionic) form in the pH range between 3 and 7, and negatively charged at pH 8–9. T-2 adsorption was also stable (69–76%) along the pH range of the study. T-2 toxin has a tetracyclic sesquiterpenoid 12,13-epoxytrichothene ring system. It is a weak basic (essentially neutral) compound with a pKa of 13.2. Therefore, hydrophobic interactions may be responsible for ZEA and T-2 adsorptions by the product in pH ranges typical of the GI tract. FB_1_ adsorption values ranged from 99 to 8% and were significantly higher when pH was ≤5. Due to the presence of carboxylic, hydroxyl, and amino functional groups in its structure, FB_1_ was the most polar mycotoxin among those tested. As discussed by Avantaggiato et al. [19], the anionic and cationic form of the toxin strongly depends on the pH, in particular the anionic form should be prevalent at pH values ≥5, and the cationic form at pH values <6. The findings of our study suggest that FB_1_ adsorption by the new binder occured mainly when pH was between 3 and 5, and electrostatic interactions or hydrogen bonds involving the carboxylic functional groups may take place. The trend of OTA adsorption is comparable to FB_1_. OTA adsorption values were 74–69% at pH 3–5, about 30% at pH 6–7, and ≤17% at pH ≥ 8. OTA is an ionizable molecule that should be in the anionic form in buffer solutions near pH 7, and in the uncharged form in acid solutions (pH < 4) [19]. Taking into account our findings, OTA adsorption seems to occur mainly when in the uncharged form and decreases when pH increased up to 7, where the anionic form is predominant. Hydrophobic interactions may drive the adsorption of OTA onto the product.

To investigate whether a pH change in the medium can produce a toxin release from the adsorbent material, a desorption study was performed as described by Greco et al. [18]. Values of mycotoxin adsorption at pH 3 and desorption at pH 7 were calculated for each toxin and expressed in percent (Table 3). Mycotoxin adsorptions were >80% for all toxins, and a change of pH did not produce a release of AFB_1_, ZEA and T-2. For these mycotoxins, desorption values were lower than 11%. Despite the low FB_1_ and OTA adsorptions recorded at pH 7 (Figure 1), the elevation of pH from 3 to 7 did not result in a total release of these toxins. Desorption values were 31% for FB_1_ and 38% for OTA. 

These findings suggest that mycotoxins can be adsorbed by the new binder in the gastric compartment of monogastric animals (at low pH) and retained during transit through the small intestine. This should prevent mycotoxin absorption at intestinal level, preserving intestinal integrity [44,45,46].

### 2.4. Effect of Adsorbent Dosage and Toxin Concentration on Adsorption of AFB_1_, ZEA, OTA, FB_1_ and T-2

**Figure 2:**
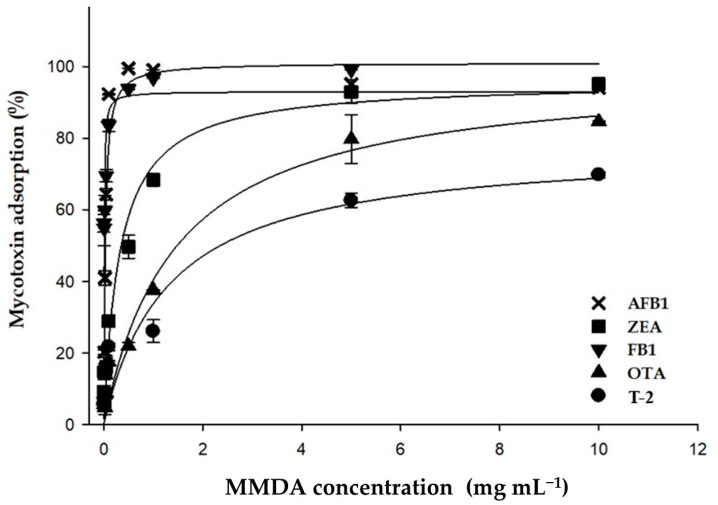
Effect of adsorbent dosage on multi-mycotoxin adsorption by a new adsorbing agent.

The effect of adsorbent dosage on simultaneous adsorption of AFB_1_, ZEA, OTA, FB_1_ and T-2 was investigated using equilibrium isotherms. Adsorption experiments were performed in triplicate, at pH 5, testing a fixed amount of toxins (1 µg mL^−1^ each) in a multi-mycotoxin solution, with different adsorbent dosages (0.005–10 mg mL^−1^). pH 5 was chosen as it represents the critical pH value above which FB_1_ and OTA adsorptions significantly dropped (Figure 1). From a biological point of view, pH 5 is the physiological pH of the duodenum compartment, where mycotoxin absorption mainly occurs. Mycotoxin adsorption data are listed in Table 4. Figure 2 shows the plots of adsorption data, expressed as percentage and plotted as a function of the adsorbent dosage. Mycotoxin adsorption was significantly affected by the dosage of the binder, and the percentage of mycotoxins removed from the multi-mycotoxins solution increased with increasing dosages. 

Experimental values of mycotoxin adsorption were in the following ranges: 9–100% AFB_1_, 56–100% FB_1_, 9–95% ZEA, 6–85% OTA, 5–70% T-2. Some DON adsorption (22%) was recorded at the higher dosages (5 and 10 mg mL^−1^). HT-2 adsorption did not occur even at these dosages.

Isotherm adsorption plots were well fitted by the Langmuir model (R^2^ > 0.990). This model allowed the calculation of two adsorption parameters, i.e., the Ads_max_ and the C_50_, which are the theoretical estimated maximum adsorption and the theoretical dosage of adsorbent, providing a 50% reduction of toxin. Ads_max_ and C_50_ calculated for AFB_1_, FB_1_, ZEA, OTA and T-2 are listed in Table 5 and expressed as percentage. C_50_ could not be calculated for DON and HT-2 since their adsorption was negligible. For all toxins, Ads_max_ and C_50_ values calculated by the Langmuir model were in accordance with the experimental values listed in Table 4. 

These results suggest a high efficacy of the composite in simultaneously adsorbing five out of the seven target mycotoxins. A dosage of 2 mg mL^−1^ should assure efficient (≥50%) and simultaneous adsorption of AFB_1_, FB_1_, ZEA, OTA and T-2 from a multi-mycotoxin solution containing 1 µg mL^−1^ of each toxin. This dosage is lower than the C_50_ values calculated for some multi-mycotoxin biosorbents tested in our previous studies [18,19], and in accordance with the findings of the two recent works using tri-octahedral bentonites as binders [12,13]. In the latter studies, some tri-octahedral bentonites when tested at 2 mg mL^−1^ adsorbed more than 90% of ZEA and FB_1_ [13], while low dosages (from 0.2 up to 2 mg mL^−1^) adsorbed 96–100% of AFB_1_ and 15–75% of OTA [12].

Equilibrium adsorption isotherms were also used to describe the effect of toxin concentration on adsorption of AFB_1_, ZEA, OTA, FB_1_ and T-2 by the new adsorbing agent. 

Adsorption is a complex process of transferring specific contaminants (such as mycotoxins) from a fluid phase to a solid phase. In order to successfully simulate and optimize the adsorption of a chosen adsorbing agent, adsorption equilibrium isotherms must be studied. The results of these studies are used to assess the affinity or capacity of an adsorbent and select a suitable adsorbent and adsorbent dose. Indeed, information obtained by these studies can also enable the estimation of the economic feasibility of an adsorbent’s commercial application for mycotoxins. 

**Figure 3:**
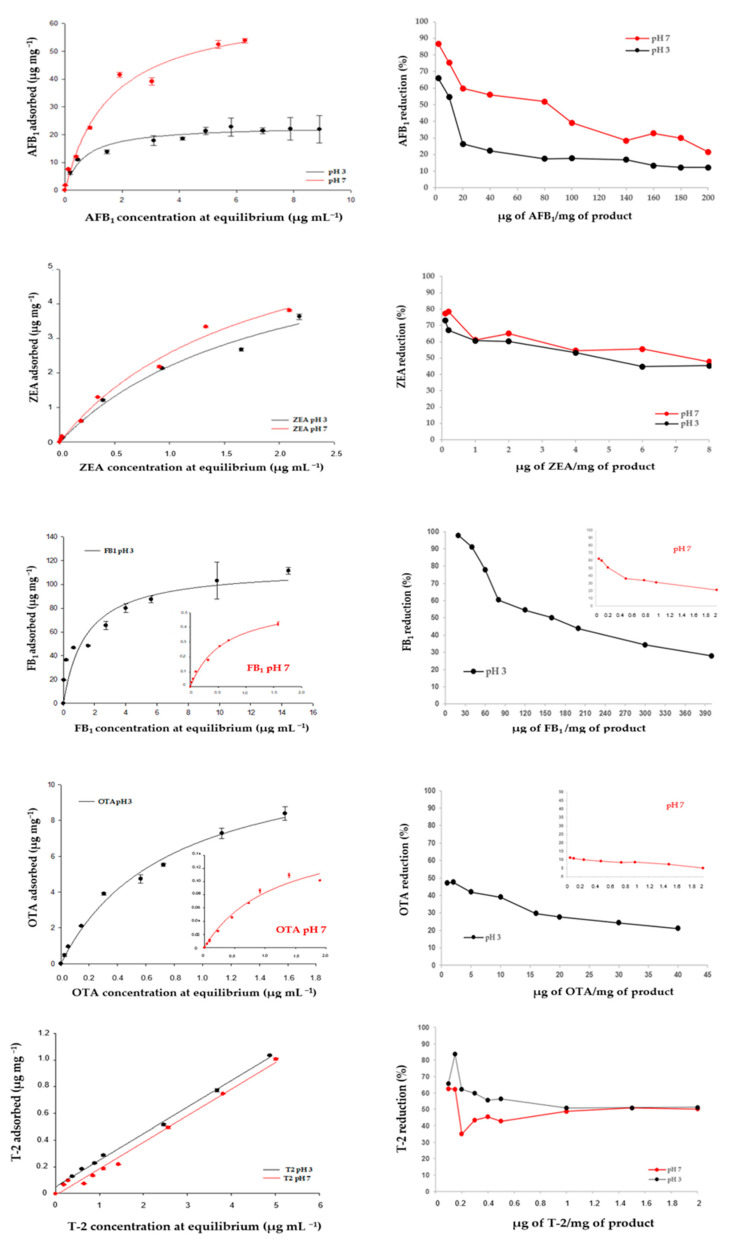
AFB_1_, ZEA, OTA, FB_1_ and T-2 adsorption isotherms obtained at constant temperature (37 °C) and pH (3 and 7) testing a fixed amount of product with increasing toxin concentrations.

Experimental adsorption data of AFB_1_, ZEA, OTA, FB_1_ and T-2 isotherms were fitted by the Freundlich, Langmuir, and Sips adsorption models to calculate the parameters involved in the adsorption process, i.e., Ads_max_ and K_L_. These parameters represent the predicted maximum adsorption capacity and the affinity of the adsorption process, respectively [19]. Most mycotoxin adsorption isotherms, excluding those obtained for T-2, were characterized by a typical L-shape (Figure 3), and were well fitted by the Langmuir model as it provided the lowest standard error statistics and the highest R^2^ values (R^2^ ≥ 0.98) compared to the other models. Adsorption parameters calculated by the Langmuir model for AFB_1_, ZEA, OTA, and FB_1_, and by the experimental adsorption values for T-2, are listed in Table 6. Following the assumptions of the Langmuir model, we can assume that the adsorption of AFB_1_, ZEA, OTA, and FB_1_ took place at definite localized sites of the adsorbing agent, which were equivalent [47]. 

The experimental values of AFB_1_ adsorption registered at pH 3 and 7 were in the ranges of 66–12% and 87–22%, respectively (Figure 3), and were obtained using an extremely low amount of product, i.e., 0.05 mg mL^−1^. The values of Ads_max_ and K_L_ differed depending on the pH of the medium (Table 6) and were higher at pH 7. Ads_max_ was 67.9 ± 2.9 µg mg^−1^ (218 ± 9 mmol Kg^−1^) at pH 7 and 24.6 ± 0.9 µg mg^−1^ (79 ± 3 mmol Kg^−1^) at pH 3. Different AFB_1_ adsorption capacities at pH 7 and 3 were also observed for the tri-octahedral smectites examined by Vila-Donat et al. [12]. K_L_ constant, related to the adsorbent affinity, was increased by decreasing the pH. At pH 3, the K_L_ was 2-fold higher compared to that calculated at pH 7, being 1.3 ± 0.3 L mg^−1^ ((41 ± 9) × 10^4^ L mol^−1^) and 0.6 ± 0.1 L mg^−1^ ((19 ± 3) × 10^4^ L mol^−1^), respectively. Experimental values of adsorption isotherms displayed in Figure 3 suggest that more than 50% of adsorption occurred when AFB_1_ and the product were in a weight ratio ≤80 µg toxin/mg of product at pH 7, and ≤20 µg toxin/mg of product at pH 3. From a practical point of view, these results suggest that more than 50% of the toxin could be adsorbed by 1 mg of the additive supplemented per 1 g of a feed (1 kg ton^−1^) containing up to 20 µg g^−1^ of AFB_1_, regardless of medium pH. This concentration is 1000 times higher than the EU-MLs (20 µg kg^−1^) and can pose serious risks in terms of livestock health and human toxicity, due to the carry-over of metabolites in food products from animals fed with aflatoxin-contaminated feed. AFB_1_ adsorption by the composite is expected to be due to smectite being the most prevalent component in the mixture. This mixture shows Ads_max_ values for AFB_1_ higher than those recorded by the study that focused on the use of tri-octahedral bentonites as mycotoxin adsorbents [12]. In the latter study, Ads_max_ values for AFB_1_ did not exceed 10 and 31 µg mg^−1^ at pH 3 and 7, respectively. However, these values are lower than those obtained by testing di-octahedral bentonites with sedimentary origin [6].

In accordance with the preliminary study, FB_1_ adsorption occurred mainly at acid pH (Figure 3). The experimental values of FB_1_ adsorption were in the ranges of 98–28% and 62–21% at pH 3 and 7, respectively, and were obtained using a dosage of 0.05 mg mL^−1^ at pH 3 and 1 mg mL^−1^ at pH 7. At these pH values, Ads_max_ was 91 ± 6 µg mg^−1^ (126 ± 8 mmol Kg^−1^) and 0.60 ± 0.02 µg mg^−1^ (0.80 ± 0.03 mmol Kg^−1^), respectively (Table 6). The Langmuir K_L_ was not pH-dependent, being 1.8 ± 0.2 L mg^−1^ ((12 ± 3) × 10^5^ L mol^−1^) and 1.6 ± 0.4 L mg^−1^ ((13 ± 1) × 10^5^ L mol^−1^) at pH 3 and 7, respectively. Taking into account the experimental values of FB_1_ adsorption (Figure 3), it can be supposed that 1 kg ton^−1^ of the product can adsorb more than 50% of the toxin in an acidic system containing up to 160 µg g^−1^ of FB_1_, a toxin concentration that exceeds the higher EU-guidance level (60 µg g^−1^) two-fold. At neutral pH, such a dosage of the product can reduce by 50% a toxin concentration lower than 0.2 µg g^−1^. Previous FB_1_ adsorption studies using tri-octahedral bentonites as binders reported lower adsorption capacities, with Ads_max_ not exceeding 50 µg mg^−1^ [13]. 

The experimental values of OTA adsorption were in the ranges of 47–21% (pH 3) and 11–5% (pH 7) and were recorded by testing the product at 0.05 and 1 mg mL^−1^, respectively (Figure 3). The respective values of Ads_max_ and K_L_ were 11.1 ± 0.4 µg mg^−1^ (28 ± 1 mmol Kg^−1^) and 1.5 ± 0.1 L mg^−1^ ((63 ± 5) × 10^4^ L mol^−1^) at pH 3; and 0.30 ± 0.03 µg mg^−1^ (0.8 ± 0.1 mmol Kg^−1^) and 0.4 ± 0.1 L mg^−1^ ((15 ± 2) × 10^4^ L mol^−1^) at pH 7 (Table 6). The experimental values of OTA adsorption isotherms and the Langmuir adsorption constants varyied depending on the pH, and allowed us to calculate the maximum toxin concentration that can be reduced by 50% by 1 kg ton^−1^ of the product. At acid pH this value was 1 µg g^−1^, which is four times higher than the higher EU-guidance level (0.25 µg g^−1^). These results show the good efficacy of the product in adsorbing OTA, and are in accordance with the findings of the preliminary study performed by Vila-Donat et al. [12]. 

At pH 3 and 7, the experimental values of ZEA adsorption were in the ranges of 73–45% and 77–48%, respectively, and were obtained by testing the adsorbent at 0.5 mg mL^−1^. Values of Ads_max_ were 4.6 ± 0.1 µg mg^−1^ (14.3 ± 0.4 mmol Kg^−1^) at pH 3 and 7.2 ± 0.5 µg mg^−1^ (23 ± 2 mmol Kg^−1^) at pH 7. These values are comparable to those recorded by Vila-Donat et al. for tri-octahedral smectites [12]. The values of ZEA adsorption affinity, K_L_, were similar when calculated at pH 3 and 7, being 0.9 ± 0.1 L mg^−1^ ((28 ± 2) × 10^4^ L/mol) and 0.5 ± 0.1 L mg^−1^ ((17 ± 2) × 10^4^ L mol^−1^), respectively. Finally, taking into account the results of adsorption isotherms, it can be calculated that 1 kg ton^−1^ of the product can bind more than 50% of ZEA with a concentration up to 4 µg g^−1^, regardless of medium pH. This toxin concentration is eight times higher than the higher EU-guidance level (0.5 µg g^−1^).

T-2 toxin adsorption was slightly affected by pH and was well described by linear adsorption isotherms (Figure 3). A linear isotherm (also known as the Henry isotherm) assumes that the concentration of adsorbate on the adsorbent surface remains constant with the adsorbate concentration, and it is usually applied to the adsorption of hydrophobic analytes in water systems. It provides no information on the monolayer adsorption capacity, in contrast to the Langmuir model. Experimental adsorption values of T-2 adsorption were in the range 66–51% at pH 3 and 62–50% at pH 7. These values were obtained testing the product at 5 mg mL^−1^ dosage. At both pH values, the maximum adsorption capacity determined experimentally was 1.0 µg mg^−1^ (2.1 mmol Kg^−1^). Isotherms in Figure 3 plotting the percentage of adsorption as a function of the weight ratio between the toxin (µg) and the product (mg) suggest that 1 kg ton^−1^ of the product can reduce by 50% a fixed toxin concentration up to 2 µg g^−1^. To date, this is the first study focused on the efficacy of a new additive containing a tri-octahedral smectite as the main component for sequestering T-2 toxin. 

The new multi-mycotoxin adsorbing agent, prepared by mixing a selected tri-octahedral smectite with a biosorbent obtained from vegetal biomasses, showed maximum adsorption capacities (Ads_max_) for target mycotoxins higher than those reported in the literature for di-octahedral smectites with hydrothermal origin, tri-octahedral smectites or biosorbents [6,12,13,18,19]. 

The overall experimental data of this in vitro study seem to support the hypothesis that the new additive can be a valuable dietary approach to remove mycotoxins from contaminated feed. The work sheds light on the additive’s mode of action and may help to interpret the results of the in vivo study recently published by our group [48]. In this preliminary study with poultry, the additive was assessed for its efficacy in counteracting the deleterious effects of an aflatoxin-contaminated diet (0.02 mg kg^−1^) supplemented with the additive at a dose of 5 kg ton^−1^ in the feed, and administered for 10 days. As described in the report published by Longobardi et al. [48], the additive reverted the nephrotoxicity induced by AFB_1_. Poultry is very sensitive to AFB_1_ intake, and oxidative stress caused by AFB_1_ plays a crucial role in chickens’ kidney damage by generating lipid peroxidation accompanied by a concomitant increase in the antioxidant enzymes involved in ROS metabolism (NADPH oxidase isoform 4 (NOX4) and its regulatory subunit p47-phox). The inclusion of the additive into the contaminated diet down-regulated both the transcription and the expression of NOX4 in chicken kidneys, together with its p47-phox subunit.

Taking into account the results of the present study, it can be suggested that the additive sequestered AFB_1_ at the GI level, thus reducing dietary exposure to AFB_1_ and counteracting oxidative stress in poultry. The antioxidant properties and the high polyphenol content in the lignin included in the final formula of the additive could have helped ameliorate the toxic effects of AFB_1_.

## 3. Conclusions 

This study provides evidence regarding the efficacy of a new feed additive acting as a multi-mycotoxin adsorbing agent. The additive was obtained by properly mixing a tri-octahedral smectite with a lignocellulose-based material. Both components were selected for their efficacy in adsorbing simultaneously most of the main occurring mycotoxins, and for the antioxidant properties of the organic component. These materials were not toxic to intestinal cells. After a simulated GI digestion, chyme samples obtained from 1 mg mL^−1^ of digested materials did not affect the viability of Caco-2TC7 cells. In addition, the chymes obtained by digesting the lignocellulose showed a high content of polyphenols (210 mg mL^−1^ catechin equivalent) and good antioxidant activity. The performance of the selected materials in removing mycotoxins was maintained in the final composite. When tested in a multi-mycotoxins system, i.e., a buffered solution at pH 5 containing a pool of mycotoxins (at 1 µg mL^−1^ each), the composite (5 mg mL^−1^ of dosage) simultaneously sequestered five out of seven assayed mycotoxins, i.e., AFB_1_ (95%), FB_1_ (99%), ZEA (93%), OTA (80%), and T-2 (63%). DON adsorption was low (22%), while HT-2 adsorption did not occur. The adsorption of AFB_1_, ZEA and T-2 was not affected by medium pH, whereas OTA and FB_1_ were adsorbed mainly at pH 3. A pH change in the adsorbing medium did not produce a release of the bound AFB_1_, ZEA and T-2 from the binder; while raising the pH from 3 to 7 desorbed 31% of FB_1_ and 38% of OTA. These findings suggest that mycotoxins can be adsorbed by the composite in the gastric compartments of monogastric animals (at low pH) and are not released during transit through the small intestine, thus preventing their absorption. Adsorption isotherm studies performed at physiological pH values showed that the product adsorbed target mycotoxins with high capacity and affinity. Maximum adsorption capacities determined at pH 7 and 3 were to 217.4 and 78.8 mmol kg^−1^ for AFB_1_; 0.8 mmol kg^−1^ and 126.1 mmol kg^−1^ for FB_1_; 22.7 mmol kg^−1^ and 14.3 mmol kg^−1^ for ZEA; 0.8 mmol kg^−1^ and 27.5 mmol kg^−1^ for OTA; and 1.1 mmol kg^−1^ and 2.1 mmol kg^−1^ for T-2. Isotherm adsorption studies allowed us to calculate the highest mycotoxin concentrations that can be reduced by 50% using a product dosage fixed at 1 kg ton^−1^. These concentrations ranged from 160 to 1 µg g^−1^ and differed depending on the toxin and pH. In all cases, these levels were higher than permitted of recommended EU limits. A dosage as low as 1 kg ton^−1^ is often used for feed additives acting as mycotoxin adsorbents and is considered safe for bentonites. 

In conclusion, the results of this study are promising and show the potential of the new additive to remove mycotoxins in practical scenarios where several mycotoxins can co-occur. Supplementation of multi-mycotoxin contaminated feeds with this additive may open new perspectives in the management of mycotoxicosis in farm animals. 

Further investigations using adsorption equilibrium isotherms, kinetics, and thermodynamics are in progress in our lab to obtain valuable information on the adsorption, the reaction pathways, and the adsorption reaction mechanisms. The effects will be evaluated of matrix components or complex mediums, such as gastric and intestinal juices, on the performances of the additive in sequestering the toxins, together with the absence of interactions with nutrients or important feed additives. As required by EFSA [49], in vivo studies using target animal species (poultry and dairy cattle) are underway to confirm the safety and efficacy of the new additive in reducing bioavailabity and toxicity of mycotoxins, thus assessing its practical utilization. The new additive will be tested at the optimal dose as determined by the present study for the adsorbed mycotoxins (AFB_1_, ZEA, OTA, FB_1_ and T-2). Results of these investigations are out of the scope of this manuscript and will be published elsewhere. 

## 4. Materials and Methods 

### 4.1. Reagents and Samples

Analytical grade chemicals were used, unless otherwise stated. All solvents (HPLC grade) were purchased from J. T. Baker (Deventer, The Netherlands). Water was of Milli-Q quality (Millipore, Bedford, MA, USA). AFB_1_, ZEA, FB_1_, OTA, DON, T-2 and HT-2 were supplied by Sigma-Aldrich (Milan, Italy). Mycotoxin adsorption was studied at different pH values using different mediums (1 mmol L^−1^), i.e., citrate buffers at pH 3, 4, and 5 and phosphate buffers at pH 6, 7, 8, and 9 [19]. AFB_1_, ZEA, OTA, DON, T-2 and HT-2 were dissolved in acetonitrile (HPLC grade). FB_1_ stock solution was prepared in acetonitrile/water (50:50, *v*/*v*). Stock solutions were stored in the dark at 4 °C. For adsorption studies, mycotoxin working solutions were prepared by properly diluting the stock solutions with the buffers into calibrated volumetric flasks. 

The materials screened in this study as multi-mycotoxin detoxifying agents, i.e., smectites (1 to 12) and lignocelluloses (1 to 6) (Table 1), were kindly provided by the Italian company New Feed Team Srl (Lodi, Italy). New Feed Team Srl also provided details about chemical composition and physicochemical properties of the materials.

Bentonite clays contained >70% smectite. Seven out of twelve smectites (items 1–7) were di-octahedral, each of the remaining items 8–12 showed a tri-octahedral structure. Except for the smectite 7 (Ca-smectite), all smectites were sodic. Smectites are phyllosilicates characterized by a sheet structure comprising layers of polyhedra of silicon oxide with tetrahedral coordination between which there is an octahedral layer. The octahedral layers contain atoms of aluminum, iron (II or III) or magnesium in their interior. When the cation of the octahedral layer is trivalent, as for example in the case of aluminum, one of every three cation positions is unoccupied; the octahedral layer has the structure of gibbsite, Al(OH)_3_, and the smectite is di-octahedral: this group includes montmorillonite, beidellite and nontronite. In tri-octahedral smectites, the cation in the octahedral layer is divalent and, as a result, all the cation positions are occupied; this gives rise to an octahedral layer with the geometry of brucite, Mg(OH)_2_, which is the case for the saponites and hectorites [4].

In order to avoid the effects of particle size on mycotoxin adsorption, all smectite samples were finely ground and sieved to obtain samples with uniform particle size. Smectite samples were analyzed for particle size by a laser diffraction particle size analyzer (Sympatec, RODOS/IM-VIBRI-HELOS/BF, Sympatec GmbH-System, Clausthal-Zellerfeld, Germany) as described by D’Ascanio et al. [6]. All samples showed uniform and fine particle size, being the parameter X_50_ (median value of the particles distribution) lower than 50 μm.

As detailed in Section 2.1 of this manuscript, the tri-octahedral smectite labelled 9 in Table 1 was selected as a multi-mycotoxin adsorbent. This smectite, in the form of a fine light beige powder (80% of particle size was below 75 μm), contained SiO_2_, Al_2_O_3_ and MgO at 54.3, 7.6 and 20.4%, respectively. Other minerals (CaO, Fe_2_O_3_, K_2_O, Na_2_O_3_, TiO_2_) were below 3%. A LOI (loss of ignition) value of 9.5% suggested that the smectite had a high degree of purity.

The lignocelluloses used were high-purity lignin products obtained as paper industry waste materials. They showed uniform and fine particle size (<200 µm). As specified by the supplier company, they were produced by using the same patented technology in different locations (India, USA and Canada), and by processing different annual fibers, such as wheat straw, sugarcane bagasse, and wood. Chemical composition and properties of these materials were comparable. They were chemically characterized as mixtures of natural polyphenols, composed of medium-to small polymer aggregates, particularly rich in OH phenolics, also containing aliphatic and carboxylic groups. 

The lignocellulose-based material listed as item 6 in Table 1 was selected for its adsorbing proprieties and antioxidant activity to prepare the formula for use in feed to remove mycotoxins. It was provided by the New Feed Team Srl as a fine light brown powder, consisting of phenylpropanoic polymer aggregates (10–12 monomeric units) with an average molar weight of around 1200–1300 g mol^−1^. The supplier indicated that the product has a high lignin content (more than 90% purity) and low content of ash (<2%) and carbohydrates (<2.5%), in particular arabinose (0.2%), galactose (0.5%), glucose (0.1%) and xylose (1.7%). It shows very low water solubility at neutral and acid pH; while under alkaline conditions, complete solubility is achieved. Furthermore, the product is poorly soluble in the most common organic solvents and stable up to 250 °C. The elemental analysis shows a high content of carbon (64.7%), oxygen (26.2%) and hydrogen (6.52%), as well as traces of nitrogen (0.52%) and sulfur (1.87%). 

As described above (see Section 2.1 and Section 2.2), the mixture containing the Na-smectite (item 9) and the lignocellulose-based material (item 6) in the weight ratio 70:30 was chosen as the best MMDA. This composite was prepared by the New Feed Team Srl, that guaranteed its homogeneity.

### 4.2. Mycotoxin Adsorption Experiments

Several materials including smectite clays (*n* = 12) and lignocelluloses (*n* = 6), and some of their mixtures, were tested for their ability in binding simultaneously some of the most frequently occurring mycotoxins from liquid buffers containing a pool of toxins, simulating gastric and intestinal pH values. 

A first batch of adsorption trials was carried out to select promising mycotoxin adsorbents to prepare the composites. These trials were performed using 1 mg mL^−1^ of adsorbent dosage and 1 µg mL^−1^ of toxin concentration, following the method of Avantaggiato et al. [19]. The mycotoxins tested for this preliminary study were AFB_1_, ZEA, OTA, FB_1_ and DON.

This study allowed the selection of two materials (i.e., the tri-octahedral smectite and the lignocellulose listed in Table 1 as items 9 and 6, respectively) as good binders for simultaneous removal of mycotoxins. These materials were properly combined, and then their mixtures were tested for simultaneous adsorption of AFB_1_, ZEA, OTA, FB_1_ and DON as they had been tested for the single components. The product obtained by mixing the smectite and the lignocellulose in the weight ratio of 70:30 was found to be the best combination.

The composite containing the smectite and the cellulose in a weight ratio of 70:30 was also tested at 1 mg mL^−1^ of dosage with a multi-mycotoxin working solution containing 1 µg mL^−1^ of AFB_1_, FB_1_, ZEA, OTA, DON, T-2, and HT-2, at pH 3, 5 and 7. With respect to the above-mentioned screening adsorption trials, T-2 and HT-2 adsorption were also evaluated. 

To perform these adsorption trials, 1 mg of each adsorbent was weighted in a 4 mL silanized amber vial and suspended with 1 mL of multi-mycotoxin buffered solution (at different pH values) containing 1 µg mL^−1^ of each mycotoxin. Adsorption experiments were carried out using an orbital shaker (KS 4000, IKA^®^-Werke GmbH & Co. KG, Staufen, Germany) set at 250 rpm, at constant temperature of 37 ± 0.5 °C. Temperature can affect toxin adsorption and should be kept constant during experiments. Preliminary trials showed fast adsorption of mycotoxins by the assayed materials, with the maximum adsorption reached in less than half an hour (data not shown). Therefore, adsorption trials were performed with 90 min of contact time to ensure the complete uptake of toxins. Overhead shakers or equivalent equipment can be used for this, and the agitation device should keep the product in suspension during shaking. After incubation, the suspension was transferred into an Eppendorf tube and centrifuged at 18,000× *g* for 20 min and 25 °C. High-speed centrifuges are advisable to remove small particles from aqueous solution. 

Blank control samples were prepared for each set of adsorption trials, using multi-mycotoxin solutions in buffer without the binders. These controls were subjected to the same test procedure and served as background control during the analysis to investigate the stability of toxins in the buffer solutions or any possible nonspecific adsorption. In addition, negative controls were prepared of buffer solutions containing the tested adsorbents without mycotoxins. These were processed identically to the test samples and used to investigate any component of materials that might interfere with chromatographic analysis of mycotoxins. All experiments, including blank and negative controls, were performed in triplicate.

At the end of the adsorption trials, supernatant samples were split into three aliquots and analysed for the residual mycotoxin content by high-performance liquid chromatography (HPLC-FLD) for FB_1_, by ultra-performance liquid chromatography (UPLC-FLD/PDA) for simultaneous detection of AFB_1_, ZEA, OTA, and DON, and by UPLC-PDA for T-2 and HT-2 detection. Aliquots of supernatant samples were appropriately diluted with buffer when necessary, then filtered by regenerated-cellulose filters (0.2 µm), transferred into glass vials and analyzed for the residual toxin content. In all cases, supernatants did not require a clean-up step prior to injection into the chromatographic systems.

### 4.3. LC Analysis of Mycotoxins

AFB_1_, ZEA, OTA and DON were simultaneously analyzed in supernatant samples of adsorption trials by an optimized UPLC method [19]. The UPLC apparatus consisted of a Waters Acquity UPLC^®^ system (Milford, MA, USA) equipped with a binary solvent manager, a sample manager with loop suitable for 1–10 μL injections, a column heater, a photodiode array detector (PDA) (Waters, Milford, MA, USA), and a spectrofluorometric detector (FLD) (Waters, Milford, MA, USA). Data acquisition and instrument control were performed by Empower 2 Software (Database Version 6.20.00.00) (Waters). The analytical column was an Acquity UPLC^®^ BEH-C18 (100 mm × 2.1 mm, 1.7 μm particle size) preceded by an Acquity UPLCTM column in-line filter (0.2 μm). Chromatographic separation of mycotoxins was achieved through 13.5 min gradient delivery of a mixture of A (water/acetonitrile, 85:15 *v*/*v*) and B (methanol/acetonitrile 50:50 *v*/*v*, containing 0.5% acetic acid) at a flow rate of 0.4 mL min^−1^. The temperatures of the column and sample manager room were maintained at 40° and 15 °C, respectively. The injected volume was 5 μL in a partial loop with needle overfill mode. 2-mL needle wash solvent (water/acetonitrile/methanol, 70:10:20 *v*/*v*/*v*) was injected after sample injection in order to avoid carry-over effects. A seal wash solution (water/acetonitrile, 90:10 *v*/*v*) was also programmed. The UV absorption spectra of mycotoxins were recorded in the range of 190–400 nm. UV absorbance data were collected with a bandwidth of 1.2 nm and without digital filtering, at wavelengths of 220 nm for DON and 350 nm for AFB_1_. For UV-analysis of these toxins, the detection wavelength was switched during the chromatographic run according to their retention time. The LC UV-chromatogram was acquired at 220 nm absorbance wavelength for the first 3 min, and then at 350 nm. For fluorescence detection of AFB_1_, ZEA and OTA, programmable wavelength switching was also used to optimize excitation and emission response, thereby improving sensitivity for individual toxins and minimizing interference. Detection was carried out using a wavelength program with excitation and emission wavelengths of 333 and 460 nm, respectively, to 7.5 min for AFB_1_ detection, then 274 and 440 nm from 7.5 to 8.5 min for ZEA, and 333 and 460 nm from 8.5 to 13.5 min for OTA. AFB_1_ was detected by PDA and FLD detectors without post-column derivatization.

T-2 and HT-2 were simultaneously analyzed by UPLC method [50]. The analytical column was an Acquity UPLC^®^ BEH-C18 (50 mm × 2.1 mm, 1.7 μm particle) preceded by an Acquity UPLCTM column in-line filter (0.2 μm). Chromatographic separation of T-2 and HT-2 was achieved through 10 min gradient delivery of a mixture of A (water) and B (acetonitrile) at a flow rate of 0.7 mL min^−1^. After elution of toxins, the column was washed by elution of 90% acetonitrile in mobile phase. This allowed shortened time of UPLC runs and cleaned the column of interfering compounds retained by the stationary phase. The temperatures of the column and sample manager room were maintained at 50 and 15 °C, respectively. The injected volume was 10 μL in a full loop with needle overfill mode. The UV absorption spectra of mycotoxins were recorded in the range of 190–350 nm. UV absorbance data were collected with a bandwidth of 1.2 nm and without digital filtering at wavelengths of 202 nm. HT-2 and T-2 retention time were 3.67 and 4.85 min respectively. Due to the high sensitivity of the UPLC analysis method, pre-column derivatization of supernatant samples was not required. The use of acetonitrile of type “gold” for LC in the mobile phase was necessary to reduce baseline drift of chromatograms at the PDA detector wavelength of 202 nm. Although UV spectra of T-2 and HT-2 toxins showed maximum adsorption at wavelengths of 191 nm and 192 nm, respectively, the wavelength of 202 nm was chosen as a good compromise between baseline drift and toxins’ sensitivity.

FB_1_ was analyzed by the HPLC-FLD method as in [19]. The HPLC-FLD apparatus was an Agilent 1100 series (Agilent, Waldbronn, Germany) equipped with a binary pump, autosampler, column thermostat set at 30 °C and a spectrofluorometric detector, with excitation and emission wavelengths set at 335 nm and 440 nm, respectively. The analytical column was a Kinetex^®^ core-shell 2.6 μm particle with pentafluorophenyl stationary phase (50 × 4.6 mm) (Phenomenex, Torrance, CA, USA). Isocratic mobile phase consisted of the water/methanol/acetonitrile mixture (50:25:25, *v*/*v*/*v*) containing acetic acid (1%), and eluted at 0.9 mL min^−1^ flow rate for 14 min. FB_1_ retention time was 7.5 min. After toxin elution, the column was washed for 3 min by 95% acetonitrile in mobile phase. Prior to HPLC analysis, FB_1_ samples were pre-column derivatized with o-phthaldialdehyde (OPA) reagent. OPA reagent was prepared by dissolving 40 mg OPA with 1 mL methanol and 5 mL sodium tetraborate (Na_2_B_4_O_7_·12H_2_O, 0.1 mol L^−1^). Then, 50 μL 2-mercaptoethanol was added and mixed for 1 min. This reagent solution was stable for up to one week at room temperature in the dark, in a capped amber vial. FB_1_ derivatization was performed by an automated pre-column derivatization programme.

LC methods of mycotoxin analysis in buffer solutions at different pH values or in aqueous supernatants of test materials were sensitive, and showed a good selectivity, accuracy and precision, with relative standard deviations (RSDs) less than 6%. Quantitative analysis of mycotoxins in supernatant samples was performed by standards calibration curves and peak-area measurement. LC methods were linear (*p* < 0.0001) in the concentration range of 0.07–1.0 μg mL^−1^ (five mycotoxin levels) for DON; 0.007–1.0 μg mL^−1^ (seven mycotoxin levels) for AFB_1_, ZEA, and OTA; 0.05–5.0 μg mL^−1^ (eight mycotoxin levels) for FB_1_; and 0.1–50 µg mL^−1^ for T-2 and HT-2 (thirteen mycotoxin levels). Calibrants were prepared in buffers at different pH values and analyzed in triplicate. The coefficients of determination (R^2^) were ≥0.996. The limits of quantitation were 70 ng mL^−1^ for DON; 7 ng mL^−1^ for ZEA; 0.7 ng mL^−1^ for OTA and AFB_1_; 50 ng mL^−1^ for FB_1_; 100 ng mL^−1^ for T-2 and HT-2 (S/N ratio = 10). These limits were 1–4 orders of magnitude below toxin concentration of the working solutions used for adsorption tests, and safeguarded the ability to perform accurate LC measurements even when strong mycotoxin adsorption occurred (>90%). These methods were selective and no compound in the aqueous supernatants of test materials interfered with the identification and quantification of mycotoxin peaks. Chemical precipitation and losses of mycotoxins due to nonspecific adsorption were not detected. Area values of LC peaks of mycotoxins for blank control samples were comparable to those for standards for aqueous supernatants of test materials spiked with toxins, at the concentrations of the standard calibration curves. Thus the methods are accurate and fitted the scope of the study.

### 4.4. Evaluation of Cytotoxicity and Antioxidant Properties of Selected MMDAs

Selected MMDAs, i.e., the trioctahedral smectite 9 and the lignocellulose-based material 6, and ascorbic acid, were subjected to a gastro-intestinal digestion process and, subsequently, the chyme sample was analysed for biological activity. Ascorbic acid was tested as a standard natural compound. Then, 400 mg of each mycotoxin adsorbent were digested following the method of Minekus et al. [51], the chyme was centrifuged (18,000× *g* for 20 min) and then an aliquot of the supernatants was diluted 10 times prior to assessment of cytotoxicity and antioxidant effect.

The cytotoxicity and cellular antioxidant activity of chyme samples was determined using the Caco-2TC7 clone, which was kindly supplied by M. Rousset (INSERM U505, Paris, France). The Caco-2TC7 clone was grown as described by Barberis et al. [52]. For cytotoxicity and antioxidant activity experiments, Caco-2TC7 cells were seeded at a starting density of 250,000 cells mL^−1^ and 500,000 cells mL^−1^, respectively, in a 96 well plate and incubated overnight to allow adhesion on the growth surface. To assess the cytotoxicity of digested mycotoxin adsorbents, the relevant chyme samples were tested on the intestinal cell line in a dose-dependent manner (from 0.0012 to 1 mg mL^−1^), after 24 h of exposure, using the MTT assay as described by Minervini et al. [53]. The antioxidant activity of chyme samples was measured as a reduction of intracellular induced Reacting Oxygen Species (ROS) applying the Cellular Antioxidant Activity (CAA) assay. The CAA assay was performed according to the procedure described by Wolfe and Liu [54]. Briefly, cellular suspension was seeded on a 96 well white flat-bottom plate and incubated at 37 °C for 24 h. After seeding, cells were stained with 5 μM of 2–7dichloro-dihydrofluorescein diacetate (DCFH-DA) and incubated for 30 min. After the staining phase, cells were treated for 30 min with chyme samples at concentrations ranging from 0.0012 to 1 mg mL^−1^. Then, cumene hydroperoxide (CMHP, 12.5 μM) was added to the cells as a stress inducer and the fluorescence (Ex 485 nm, Em 530 nm) was measured every 5 min for 1 h at 37 °C using a Varioskan Flash Spectral Scanning Multimode Reader (Thermo Fisher Scientific, Milan, Italy). This procedure allowed the mathematical calculation of different parameters, including the CAA Unit and the Median effective Dose (EC_50_) as described by Garbetta et al. [55]. The CAA unit was measured by integrating the area under the kinetic curve (fluorescence vs. time). Higher values of CAA units indicate a high antioxidant activity. The EC_50_ is the concentration of material (mg mL^−1^) that produces a 50% reduction of induced ROS. 

In addition, the total polyphenol content was measured by the Folin-Ciocalteau (FC) assay following the method of Singleton and Rossi [56]. In particular, 200 μL of the digested sample were combined with 500 μL of FC reagent (containing phosphomolybdic/phosphotungstic acid complex) and incubated in alkaline conditions with a Na_2_CO_3_ solution (20% *w*/*v*) for 20 min. At the end of incubation, the absorbance value was measured at 750 nm and the polyphenol concentration was calculated by a standard curve obtained by linear regression analysis of catechin and chlorogenic acid as standards.

### 4.5. Effect of pH and Desorption Study 

To study the effect of pH on multi-mycotoxin adsorption by the composite, triplicate independent experiments were performed at pH values in the range 3–9, using an adsorbent dosage fixed at 5 mg mL^−1^ and a toxin concentration of 1 μg mL^−1^ for each mycotoxin in the pool (i.e., AFB_1_, ZEA, OTA, FB_1_, DON, T-2 and HT-2). 

The desorption study was performed following Avantaggiato et al. [19]. In this regard, 5 mg of the mixture were weighed into a 2 mL screw-cap test tube and mixed with 1 mL of the multi-mycotoxin working solution (pH 3), containing 1 μg mL^−1^ of the toxins (AFB_1_, ZEA, OTA, FB_1_, DON, T-2 and HT-2). Samples were incubated at 37 °C for 90 min in an orbital shaker set at 250 rpm. They were then centrifuged, and the supernatants were completely removed and analysed for residual mycotoxin content to calculate mycotoxin adsorption. Remaining adsorbent pellets were washed with 1 mL of buffer solution at pH 7, incubated for 30 min in an orbital shaker set at 37 °C and 250 rpm, and then centrifuged to separate the supernatants for mycotoxin analysis and mycotoxin desorption assessment. Desorption values, expressed as percentages, were reported as the mean of three independent replicates. 

### 4.6. Equilibrium Adsorption Isotherms

The effect of adsorbent dosage and mycotoxin concentration on adsorption of AFB_1_, ZEA, OTA, FB_1_ and T-2 by the mixture was studied by equilibrium adsorption isotherms according to Avantaggiato et al. [18]. DON and HT-2 were excluded from this study since their adsorption was negligible, as indicated by the preliminary adsorption experiments. Two different sets of adsorption isotherms were performed by triplicate independent experiments. 

The first set of isotherms allowed the assessment of the effect of the amount of adsorbent material on the simultaneous adsorption of AFB_1_, ZEA, OTA, FB_1_ and T-2. It was performed at constant pH (pH 5) and temperature (37 °C), testing simultaneously the pool of mycotoxins (1 µg mL^−1^ each) with different adsorbent dosages (0.005–10 mg mL^−1^). 

The second set of isotherms was aimed at determining the effect of toxin concentration on adsorption, and was performed at constant temperature (37 °C) and pH (pH 7 and pH 3) testing a fixed amount of adsorbent towards standard solutions containing increasing mycotoxin concentrations. In the second set of adsorption isotherms, mycotoxins were assayed individually. These isotherms were useful for the calculation of certain parameters related to the adsorption process, including maximum adsorption capacity Ads_max_, and adsorption affinity (K_L_). Preliminary adsorption trials were performed under the same experimental conditions to determine the proper adsorbent material/mycotoxin solution ratio, and also the range of toxin concentrations to be used in the study (data not shown). Mycotoxin/adsorbent combinations providing adsorption values ranging from 100 to <50% were considered optimal for the adsorption isotherms. Due to different abilities in sequestering structurally different mycotoxins, the MMDA dosage ranged from 0.05 to 5 mg mL^−1^ depending on the toxin. In particular, a low dosage (0.05 mg mL^−1^) was used for testing the MMDA with AFB_1_ (at both pH values), FB_1_, and OTA at pH 3. A 1 mg mL^−1^ dosage was used for FB_1_ and OTA at pH 7. ZEA isotherms were obtained testing the MMDA at 0.5 mg mL^−1^ dosage. A 5 mg mL^−1^ dosage was used in the case of testing the MMDA with T-2 at both pH values. Mycotoxin concentrations were in the range 0.05–10 µg mL^−1^ for AFB_1_, 0.05–4 µg mL^−1^ for ZEA, 0.05–2 µg mL^−1^ for OTA, 1–20 µg mL^−1^ for FB_1_, and 0.5–10 µg mL^−1^ for T-2 and HT-2. These concentrations did not pose any problems for mycotoxin solubility in water, and were high enough to allow the determination of the experimental adsorption values. 

### 4.7. Data Calculations and Curve Fitting

The amount of bound mycotoxin was calculated as the difference between the amount of mycotoxin in the supernatant of the blank tubes with no test product and the amount found in the supernatant of the experimental tubes with the adsorbents. This amount was compared to the quantity present in the supernatant of the blank tubes, and was expressed in percent. 

Concerning the isotherms adsorption study, the first set of adsorption isotherms was obtained by plotting the percentage of mycotoxin adsorbed (%_ads_) as a function of adsorbent dosage, %_ads_ = *f* (dosage). The second set of adsorption isotherms was obtained by plotting the amount of mycotoxin adsorbed per unit of mass of product Q_eq_ against the concentration of the toxin in the external phase C_eq_, under equilibrium conditions, Q_eq_ = *f*(C_eq_). The experimental adsorption values obtained by the second set of isotherms were expressed as percentages and plotted against the weight ratio between the mycotoxin and the adsorbing product [µg mycotoxin/mg product]. Experimental adsorption data were calculated, plotted, transferred to SigmaPlot (Systat.com, version 12.0), and then fitted by either the linear isotherm model or the multiple isotherm equations, i.e., the Langmuir, Freundlich and Sips equations. The model that fitted the experimental data most accurately was used to describe the system and predict the adsorption behavior [19]. 

For both isotherm sets, each experimental point in the plots represents the mean of three independent replicates.

## Figures and Tables

**Table 1:** Simultaneous adsorption of AFB_1_, ZEA, OTA, and FB_1_ recorded for lignocellulose-based materials and clays (di- or tri- octahedral smectites) tested at constant temperature (37 °C), for 90 min contact time, at different pH values. The dosage of materials was 1 mg mL^−1^. The multi-mycotoxin solution contained 1 μg mL^−1^ of AFB_1_, ZEA, OTA, FB_1_ and DON. Values are means of triplicate independent experiments. DON adsorption values were negligible and are not shown.

Materials	Mycotoxin Adsorption (%)							
	AFB_1_		ZEA		OTA		FB_1_	
	pH 3	pH 7	pH 3	pH 7	pH 3	pH 7	pH 3	pH 7
Lignocellulose 1	76	82	67	70	73	1	23	3
Lignocellulose 2	70	82	49	61	56	2	19	8
Lignocellulose 3	79	70	76	67	66	0	23	3
Lignocellulose 4	59	60	61	63	62	3	0	7
Lignocellulose 5	47	87	37	68	35	0	25	8
Lignocellulose 6	81	71	77	71	72	0	39	6
Na-smectite 1 (di-octahedral)	99	97	4	7	29	2	92	9
Na-smectite 2 (di-octahedral)	87	84	15	6	1	1	27	1
Na-smectite 3 (di-octahedral)	100	100	18	4	55	3	100	8
Na-smectite 4 (di-octahedral)	100	98	28	8	74	3	96	8
Na-smectite 5 (di-octahedral)	100	100	34	14	77	4	100	4
Na-smectite 6 (di-octahedral)	100	99	38	24	75	1	99	8
Ca-smectite 7 (di-octahedral)	100	100	36	20	71	2	100	20
Na-smectite 8 (tri-octahedral)	99	83	2	3	10	0	88	6
Na-smectite 9 (tri-octahedral)	98	98	77	81	90	14	100	26
Na-smectite 10 (tri-octahedral)	99	99	79	77	88	6	100	27
Na-smectite 11 (tri-octahedral)	100	99	84	76	77	4	100	13
Na-smectite 12 (tri-octahedral)	100	99	89	82	80	25	99	25

**Table 2:** Multi-mycotoxin adsorption efficacy of a mixture containing a tri-octahedral smectite and a lignocellulose-based material in the weight ratio of 70:30. The mixture at 1 mg mL^−1^ of dosage was tested at constant temperature (37 °C), for 90 min contact time, at different pH values. The multi-mycotoxin solution contained 1 μg mL^−1^ of AFB_1_, ZEA, OTA, FB_1_, DON, T-2 and HT-2. Adsorption values are means (±SD) of triplicate independent experiments.

pH	Mycotoxin Adsorption (%)						
	AFB_1_	FB_1_	ZEA	OTA	T-2	DON	HT-2
7	100.0 ± 0.0	1.5 ± 1.3	51.5 ± 0.0	2.8 ± 0.3	33.4 ± 2.9	8.1 ± 0.8	0.0 ± 0.0
5	96.7 ± 1.4	91.0 ± 0.9	64.5 ± 0.2	31.9 ± 0.6	23.4 ± 1.6	0.5 ± 0.4	0.0 ± 0.0
3	100.0 ± 0.0	94.2 ± 0.9	65.8 ± 0.7	84.0 ± 0.5	44.9 ± 0.8	13.6 ± 1.3	0.0 ± 0.0

**Table 3:** Multi-mycotoxin adsorption and desorption values. Adsorption tests were performed at pH 3, using an adsorbent dosage of 5 mg mL^−1^ and a toxin concentration of 1 µg mL^−1^. Desorption values were determined at pH 7. Values are means ± standard deviations of triplicate independent experiments.

Toxin	Adsorption pH 3 (%)	Desorption pH 7 (%)
AFB_1_	96.6 ± 0.6	1.7 ± 0.3
ZEA	91.6 ± 0.0	4.1 ± 0.7
FB_1_	99.6 ± 0.1	30.6 ± 2.7
OTA	91.7 ± 0.4	38.1 ± 4.6
T-2	81.2± 1.6	10.6 ± 0.2

**Table 4:** Effect of adsorbent dosage on simultaneous multi-mycotoxin adsorption by a mixture containing a tri-octahedral smectite and a lignocellulose-based material. Adsorption tests were performed at pH 5, using an adsorbent dosage ranging from 0.005 to 10 mg mL^−1^, and 1 µg mL^−1^ of toxin concentration. Values are means ± standard deviations of triplicate independent experiments.

Dosage (mg mL^−1^)	Mycotoxin Adsorption (%)						
	AFB_1_	FB_1_	ZEA	OTA	DON	T-2	HT-2
0.005	8.5 ± 2.3	56.2 ± 2.5	9.2 ± 0.8	5.6 ± 0.1	0.7 ± 1.0	5.7 ± 1.6	2.5 ± 1.6
0.01	20.2 ± 0.6	54.8 ± 4.9	14.7 ± 2.2	8.6 ± 0.7	4.4 ± 3.0	7.5± 3.4	6.7± 3.4
0.025	40.9 ± 2.0	59.8 ± 4.1	14.5 ± 1.2	4.7 ± 2.0	2.8 ± 1.4	5.9 ± 0.8	2.9 ± 0.8
0.05	64.3 ± 1.5	69.5 ± 1.8	16.1 ± 3.1	8.7 ± 1.5	4.9 ± 0.8	15.7 ± 3.0	2.1 ± 3.0
0.1	92.3 ± 0.3	83.6 ± 1.8	28.8 ± 0.9	17.5 ± 0.4	11.0 ± 0.1	21.7 ± 1.0	11.1 ± 1.0
0.5	99.5 ± 0.0	93.8 ± 0.2	49.7 ± 3.2	21.9 ± 1.0	3.9 ± 0.5	11.7 ± 1.3	0.0 ± 0.0
1	99.1 ± 0.0	96.8 ± 0.2	68.3 ± 0.6	37.6 ± 0.1	8.6 ± 0.4	26.0 ± 3.2	0.0 ± 0.0
5	95.1 ± 1.2	99.1 ± 0.1	92.9 ± 3.3	79.7 ± 6.8	21.9 ± 0.6	62.6 ± 2.0	0.0 ± 0.0
10	94.1 ± 0.3	99.5 ± 0.1	95.1 ± 0.1	84.6 ± 0.2	22.0 ± 0.6	69.7 ± 0.9	0.0 ± 0.0

**Table 5:** Theoretically estimated values for maximum adsorption (Ads_max_) and inclusion rate (mg mL^−1^) of the adsorbing agent to obtain a 50% reduction of the absorbable toxin (C_50_) ^a^.

Toxin	Ads_max_	C_50_
AFB_1_	103 ± 3	0.03
FB_1_	96 ± 3	0.01
ZEA	96 ± 6	0.3
OTA	99 ± 8	1.5
T-2	78 ± 9	2.3

^a^ Ads_max_ and C_50_ were calculated using the Langmuir isotherm model.

**Table 6:** Isotherm model parameters for the adsorption of different mycotoxins by a new multi-mycotoxin adsorbing agent prepared by mixing a selected tri-octahedral smectite with a biosorbent. Isotherm parameters were obtained at 37 °C, pH 7 and 3, and were calculated by the Langmuir model for most mycotoxins. For T-2, the Ads_max_ values represent the maximum experimental adsorption values.

Adsorption Parameters	AFB_1_		AFB_1_		ZEA		OTA		T-2	
	pH 7	pH 3	pH 7	pH 3	pH 7	pH 3	pH 7	pH 3	pH 7	pH 3
Ads_max_^1^	67.9	24.6	0.6	91.0	7.2	4.6	0.3	11.1	1.0	1.0
K_L_^2^	0.6	1.3	1.8	1.6	0.5	0.9	0.4	1.6	-	-
Dosage ^3^	0.05	0.05	1	0.05	0.5	0.5	1	0.05	5	5

^1^ Maximum binding capacity (µg mg^−1^); ^2^ Adsorption affinity (L mg^−1^); ^3^ Adsorbent dosage used to carry out the equilibrium adsorption isotherms (mg mL^−1^).

## Data Availability

Not applicable.
